# Experiences, acceptability and barriers to calcium supplementation during pregnancy in Dar es Salaam, Tanzania

**DOI:** 10.1111/mcn.13732

**Published:** 2024-09-24

**Authors:** Emmy O. Metta, Alfa Muhihi, Heavenlight A. Paulo, Christopher R. Sudfeld, Anna Kaale, Nandita Perumal, Mary Mwanyika‐Sando, Ndeniria O. Swai, Christopher P. Duggan, Honorati Masanja, Blair J. Wylie, Andrea B. Pembe, Wafaie Fawzi

**Affiliations:** ^1^ Department of Behavioral Sciences Muhimbili University of Health and Allied Science Dar es Salaam Tanzania; ^2^ Africa Academy for Public Health Dar es Salaam Tanzania; ^3^ Department of Epidemiology and Biostatistics Muhimbili University of Health and Allied Science Dar es Salaam Tanzania; ^4^ Department of Global Health and Population Harvard T.H. Chan School of Public Health Boston Massachusetts USA; ^5^ Department of Nutrition Harvard T.H. Chan School of Public Health Boston Massachusetts USA; ^6^ Ifakara Health Institute Dar es Salaam Tanzania; ^7^ Department of Epidemiology and Biostatistics, Arnold School of Public Health University of South Carolina Columbia South Carolina USA; ^8^ Dar es Salaam Regional Medical Office of Health Dar es Salaam Tanzania; ^9^ Division of Gastroenterology, Hepatology and Nutrition Boston Children's Hospital Boston Massachusetts USA; ^10^ Department of Obstetrics and Gynecology Columbia University Medical Center New York New York USA; ^11^ Department of Obstetrics and Gynecology Muhimbili University of Health and Allied Science Dar es Salaam Tanzania

**Keywords:** calcium, pregnancy, prenatal care, prenatal supplements, qualitative research

## Abstract

Calcium supplementation in pregnancy is recommended in contexts with low dietary calcium intake to reduce the risk of pre‐eclampsia and its complications. The World Health Organisation suggested high‐dose calcium supplementation (1500–2000 mg/day), divided into three doses and taken at different times from daily iron‐folic supplements. We conducted a mixed methods evaluation study to assess experiences, acceptability and barriers to high‐dose calcium supplementation from the perspectives of pregnant women and antenatal health care providers at two public health facilities in Dar es Salaam, Tanzania. Descriptive statistics and thematic analysis were used to characterise acceptability, barriers and overall experiences of using high‐dose calcium supplementation. Pregnant women in the cohort were aged 19–41 years, with 32.4% being primiparous. The proportion of pregnant women who liked calcium supplements ‘a lot’ decreased from 50.2% at the first visit to 31.8% at the last antenatal follow‐up visit. Adherence was 71.3% (interquartile range: 50.5%, 89.3%), with only 24.0% of the participants taking 90% or more of the required supplements. Although participants expressed positive attitudes towards using calcium supplements, they also voiced concerns about the large size, side effects, the potential to forget and the burden of taking calcium supplements three times per day. Antenatal health care providers also affirmed the high burden of taking calcium supplements in addition to iron‐folic acid supplements. Participants expressed the acceptability of using calcium supplements during pregnancy, but adherence to three doses per day posed challenges to pregnant women. Reducing the number of calcium supplement doses per day may improve adherence.

## Key messages

- Context‐specific data regarding acceptability and adherence is important for successful implementation, uptake and scale‐up of nutritional interventions.
- High‐dose antenatal calcium supplementation was acceptable. However, adherence to three doses per day was challenging for pregnant women. Barriers included large size, side effects, potential to forget and burden of taking three doses calcium supplements per day in addition to iron‐folic supplements.
- Reducing calcium supplements pill burden during pregnancy may reduce programme costs, improve adherence and maximise potential benefits of the intervention. Scale up of anetanatal calcium supplementation should build on existing acceptability and adherence data.

## Graphical abstract

We assessed experiences, acceptability and barriers to higher‐dose calcium supplementation from the perspectives of pregnant women and antenatal health care providers in Dar es Salaam, Tanzania. Calcium supplementation was acceptable to pregnant women. However, they voiced concerns about large size, side effects, potential to forget and burden of taking three doses per day.

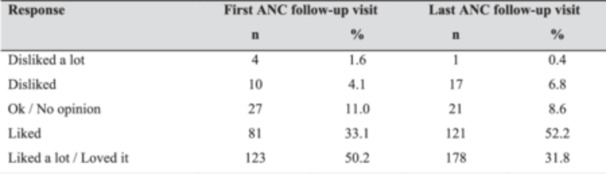

## 1 INTRODUCTION

Hypertensive disorders of pregnancy (HDPs) are among the leading direct causes of maternal and prenatal morbidity and mortality globally (Behrens et al., 2017; Chappell et al., 2021; Huang et al., 2022). HDPs, which include chronic hypertension, gestational hypertension, pre‐eclampsia and chronic hypertension with superimposed pre‐eclampsia account for 14% of maternal deaths globally (Say et al., 2014). The majority of the deaths occur in low‐ and middle‐income countries (LMICs), where the prevalence of HDP ranges from 1.8% to 16.7% (Moshi & Tungaraza, 2021). A systematic review of studies published between 2015 and 2020 found HDPs to be the second leading cause of maternal mortality accounting for 22.1% of maternal deaths in Sub‐Saharan Africa (Musarandega et al., 2021). In Tanzania, pre‐eclampsia/eclampsia accounted for 19.9% of all maternal deaths (Pembe et al., 2014). There is also well‐established literature on the association between HDPs and pre‐term birth, the leading cause of mortality in children under 5 years of age, globally (Ahmed et al., 2023; Hofmeyr et al., 2018; Kassebaum et al., 2017)

Evidence from randomised controlled trials shows that calcium supplementation during pregnancy prevents pre‐eclampsia and pre‐term birth (Hofmeyr et al., 2018). As a result, the World Health Organisation (WHO) recommends high‐dose calcium supplementation for pregnant women in populations with low dietary calcium intake to reduce the risk of pre‐eclampsia (WHO, 2018). The recommended dose is 1500–2000 mg of calcium daily, divided into three doses, preferably each taken at mealtimes and separately from iron‐folic acid (IFA) supplements.

Much of the morbidity and mortality associated with pre‐eclampsia and eclampsia could be avoided by implementing proven interventions (Hofmeyr et al., 2018). However, the practical aspects of implementing such a complex dosing schedule in programmatic settings, especially in populations where anaemia is a public health problem are not well documented. The success of such interventions depends on many aspects, including the acceptability of the interventions by antenatal health care providers and pregnant women. Thus, it is imperative to understand the context informing the implementation of proven interventions for primary prevention of HDPs. Like many other LMICs, Tanzania has not yet implemented routine calcium supplementation in pregnancy per WHO recommendation.

We conducted a mixed‐methods evaluation study of a cohort of pregnant women who received standard‐of‐care counselling for high‐dose calcium supplementation at two public health facilities in Dar es Salaam Tanzania. We used the theoretical framework of acceptability to assess acceptability and identify barriers to calcium supplementation among pregnant women and health care providers.

## 2 METHODS

### 2.1 Study design and setting

This mixed methods study was conducted at two public health facilities in Dar es Salaam, Tanzania. The two public health facilities were purposely selected from two municipalities (Ubungo and Temeke) and they were clinics that had high numbers of antenatal care clinic (ANC) attendees per year. We recruited participants for the pregnancy cohort, as a separate component of a trial to assess the non‐inferiority of low‐dose compared to standard high‐dose calcium supplementation among 22,000 pregnant women in India and Tanzania. The trial protocol, including a description of the study sites, was published elsewhere (Dwarkanath et al., 2021).

### 2.2 Pregnant women

#### 2.2.1 Pregnancy cohort

Participants for the pregnancy cohort were enroled at two public health facilities, separate from health facilities for the individual randomised clinical trial. Participants were adults aged 18 years and above, attended ANC for the first time at ≤20 weeks gestation (based on the date of the last normal menstrual period), intended to stay in the study area until 6 weeks post‐partum, and provided written informed consent. Pregnant women with a history, signs or symptoms of nephrolithiasis, history of parathyroid disorder or a thyroidectomy or a disease requiring digoxin, phenytoin or tetracycline therapy were excluded. Participants also consented to follow‐up visits and assessments including participating in in‐depth interviews (IDIs) and/or focus group discussions (FGDs). A total of 250 pregnant women were enroled in the pregnancy cohort and received standard‐of‐care counselling on calcium supplementation. The calcium counselling session covered information regarding the health benefits of calcium supplementation to both the mother and unborn child, anticipated minor side effects of calcium supplementation, how and when to take calcium supplements during the day, including taking calcium and iron‐folic supplements at least 2 h apart, and what to do in case they forgot to take the supplements. All participants received a 35‐day supply of open‐labelled calcium supplements, and calcium refills during each subsequent routine ANC clinic visit until delivery.

#### 2.2.2 FGDs and IDIs

A sub‐group of participants in the pregnancy cohort was purposely selected to participate in FGDs and IDIs. Participants for FGDs and IDIs were selected from a list of participants who were between 32 and 36 completed weeks of gestation. Selection criteria included age (young women and older women), gravidity (primigravida and multi‐gravida), duration in the study (<15 weeks and more than 15 weeks), ANC attendance (<4 visits and ≥4 visits), marital status (single and married), education level (primary, secondary and above). We conducted separate FGDs for primigravida and multi‐gravida, and a mixed FGD comprising both primigravida and multi‐gravida. A total of 52 pregnant women participated in FGDs (44) and IDIs (8).

### 2.3 Antenatal health care providers

We conducted six IDIs with antenatal health care providers who provided antenatal care services to pregnant women enroled in the pregnancy cohort at the two public health facilities. IDIs with antenatal health care providers aimed at understanding their experiences in providing health education, counselling and calcium supplements to pregnant women.

### 2.4 Data collection

#### 2.4.1 Quantitative data collection

At the first ANC visit, study staff collected information regarding sociodemographic and economic information, pregnancy history, history of hypertension and recent medical history. During each follow‐up ANC visit, blood pressure and proteinuria were checked and documented. Adherence to the thrice daily calcium supplementation was assessed objectively using pill count. Study staff documented the number of calcium pills given and the number of pills returned at each ANC visit. Participants also self‐reported whether they liked using calcium supplements on a 5‐point Likert scale ranging from ‘Disliked a lot’ to ‘Liked a lot’. Reasons for missing calcium supplements were also documented.

#### 2.4.2 Qualitative data collection

A total of six FGDs (pregnant women), eight IDIs (pregnant women) and six IDIs (health care providers) were conducted. The FGDs aimed at exploring women's perspectives and opinions about calcium supplementation and its acceptability in general during pregnancy. The IDIs aimed at understanding specific participant's attitudes, opinions and experiences about calcium and its acceptability. The IDIs and FGDs were conducted in a private room at the health facilities. All interviews and discussions were conducted in Kiswahili (a common language for researchers and participants). Data collection guides were piloted outside the study clinics, and findings were used to refine the final guides used for data collection. FGDs lasted between 1.5 and 2 h and IDIs 1 and 1.5 h.

### 2.5 Data management and analysis

Quantitative data was processed and analysed using Stata version 17.0 (Stata Corp.). Descriptive statistics were used to characterise the study participants. Adherence was defined as the number of calcium pills consumed divided by the number of calcium pills the participant was expected to consume for the duration of participation in the study multiplied by 100. Participants who forgot to bring the calcium supplement boxes back to the clinic were assumed to have taken all the doses. Adherence was compared with self‐reports of how participants liked using calcium supplements for better understanding and interpretation of the data.

All FGDs and IDIs were audio recorded, transcribed verbatim into Kiswahili word files within 24 h, and then translated into English. The senior social scientist (E. O. M.) cross‐checked the transcriptions and translations for clarity and quality assurance before importing them to NVivo 12 (QSR International Pty Ltd.) for management and analysis. Data analysis followed six steps of qualitative data analysis as described by Braun and Clarke et al. (2006). Step 1: Familiarisation with data which involved active and repeated reading while searching for meanings and patterns in the data. Step 2: Generating initial data‐driven codes (inductive and deductive) and organising data into meaningful groups, which was done by E. O. M. and A. K. Discrepancies were resolved through discussions. Step 3: Sorting the codes into potential themes and collating the relevant coded text into the identified themes. Step 4: Review of identified themes to ensure coherence and meaning and a clear distinction between them. Step 5: Defining and refining the themes that emerged to ensure clarity and understanding of the aspects entailed and linking them with the constructs of the theoretical framework of acceptability. Step 6: Write the manuscript, describing the findings based on the theoretical framework of acceptability developed by Sekhon et al. (2017).

### 2.6 Ethics statement

The study received ethical approval from Harvard T.H. Chan School of Public Health (IRB17‐1874), Ifakara Health Institute (IHI/IRB/No: 002‐2018), Muhimbili University of Health and Allied Sciences (MUHAS‐REC‐3‐2020‐107), National Institute for Medical Research (NIMR/HQ/R.8a/Vol. IX/2752) and Tanzania Medicines and Medical Device Authority (TFDA0018/CTR/0010/5). Written informed consent was obtained from all participants before participation.

## 3 RESULTS

### 3.1 Characteristics of participants in the pregnancy cohort

A total of 250 pregnant women were enroled in the pregnancy cohort, of whom 52 (20.8%) participated in the qualitative study component, including 44 who participated in the FGDs and eight in the IDIs. The pregnant women's ages ranged from 19 years to 41 years, with the majority being 18–24 years. Most were married (89.6%) and had completed primary or secondary education (86.4%) (Table 1).

**Table 1:** Baseline characteristics of study participants.

Participant characteristics	All participants in the pregnancy cohort *n* (%)	Pregnant participants for qualitative *n* (%)
Maternal age		
18–24 years	115 (46.0)	23 (44.2)
25–29 years	64 (25.6)	12 (23.1)
30+ years	71 (28.4)	17 (32.7)
Maternal education		
No formal education	9 (3.6)	–
Primary education	121 (48.4)	20 (38.5)
Secondary education	95 (38.0)	27 (51.9)
Higher education	25 (10.0)	5 (9.6)
Marital status		
Never married	25 (10.0)	8 (15.4)
Married or living together	224 (89.6)	44 (84.6)
Widowed	1 (0.4)	–
Gravidity		
Primigravida	81 (32.4)	18 (34.6)
Gravida 2 or 3	137 (54.8)	30 (57.7)
Gravida 4+	32 (12.8)	4 (7.7)
Number of previous livebirths		
None	101 (40.4)	24 (46.2)
1 or 2	123 (49.2)	25 (48.1)
3+	26 (10.4)	3 (5.7)

### 3.2 Characteristics of the antenatal health care providers

A total of six antenatal health care providers participated in the qualitative study. They included registered nurses (2), enroled nurses (2), an assistant nurse officer (1) and a medical attendant (1). The age of the health care providers ranged from 28 to 54 years, and they had either a certificate or diploma in nursing. One health care provider had 5 years of experience while the others each had more than 10 years of working experience, mostly in providing reproductive, maternal and newborn health services.

### 3.3 Quantitative findings

#### 3.3.1 Calcium supplements use

Table 2 below shows the self‐reported acceptability of calcium supplements among pregnant women at their first and last ANC visit. Although the proportion of participants who reported liking the calcium supplements (Likert scale 4 or 5) remained relatively unchanged, pregnant women who reported liking calcium supplements ‘a lot’ decreased from 50.2% during the first ANC follow‐up visit to 31.8% at the last ANC follow‐up visit. Participants self‐reported missing taking calcium supplements because of forgetting, unpleasant smell, unpalatable taste, upset stomach or large size of the supplements at few ANC follow‐up visits (51/871, 5.8%).

**Table 2:** Self‐report Likert scale of the acceptability of calcium supplements.

	**First ANC follow‐up visit**		**Last ANC follow‐up visit**	
**Response**	***n***	**%**	***n***	**%**
Disliked a lot	4	1.6	1	0.4
Disliked	10	4.1	17	6.8
Ok/no opinion	27	11.0	21	8.6
Liked	81	33.1	121	52.2
Liked a lot/loved it	123	50.2	178	31.8

#### 3.3.2 Adherence to calcium supplements

The median adherence assessed by pill count was 71.3% (IQR = 50.5%, 89.3%). Only 24.0% of the pregnant women took ≥90% of the calcium supplements they were expected to have taken and only 44.8% took ≥75% of the calcium supplements they were expected to have taken for their duration in the study. Approximately a quarter of the participants (24.8%) took ≤50% of the calcium supplements they were required to have taken (Table 3).

**Table 3:** Adherence to calcium supplements among pregnant participants.

Percent pills taken Median (Q1, Q3)	71.3% (50.5%, 89.3%)
Percent of participants taking ≥90% of pills	24.0%
Percent of participants taking ≥75% of pills	44.8%
Percent of participants taking >50% of pills	75.2%
Percent of participants taking ≤50% of pills	24.8%

### 3.4 Qualitative findings

Qualitative findings are presented under the seven constructs of the theoretical framework of acceptability.

#### 3.4.1 Affective attitude

Participants expressed positive attitudes towards calcium supplements. It was reported that calcium supplements were good for the health of both the mother and her baby. Participants said that they were given calcium supplements to prevent them from getting eclampsia and to prevent their babies from being born prematurely.

‘Calcium supplements are good because they help to prevent eclampsia and strengthen the body … you become strong even when you come to give birth you give birth safely without any problem’.

FGD‐02, 23 years, primigravida.

#### 3.4.2 Burden

Participants described the burden experienced during the use of calcium supplements concerning the large size of calcium supplements which posed difficulties during swallowing and the number of supplements that participants were required to take per day.

i) ***General burden related to taking calcium supplements:***
  Many participants expressed concerns regarding the large size of the calcium supplements and the risk of choking.
  ‘The size is frightening … when I first received them, I was scared. Is it like this? When you see them, they are terrifying. I was almost not going to use them, but after thinking, I said, let me listen to the nurse and use them’.
  FGD‐03, 31 years, Multi‐gravida
  Participants reported undertaking a variety of strategies to enable them to take calcium supplements. Some mentioned crushing the supplement into two halves and taking one half after the other. Others reported chewing or dissolving the supplement into liquids
  ‘It is difficult to swallow it because the supplement is big; when I swallow, it does not go through; it chokes me even if I take it with much water, it still does not go easily. So, I must crush it in half before swallowing’.
  IDI‐092, 21 years, Primigravida
  ‘For me, when I started using them, I could feel it sitting here (pointing at her throat),…’.
  FGD‐05, 24 years, primigravida
  Concerns about the large size of the calcium supplements were also raised during interviews with ANC health care providers.
  ‘The calcium supplement looks large; mothers complain a lot about this. I see this as a reason for mothers not taking the supplements well’.
  ANC health care provider
ii) ***Burden related to three pills per day:***
  Pregnant women mentioned that the number of calcium supplements they had to take per day was a challenge because they also still had to take IFA supplements. As a result, taking both calcium supplements and iron‐folic supplements concurrently, or without hours apart were common reported practices. They reported getting tired of the many tablets.
  ‘You know the calcium supplements we use are many … I mean we take three calcium supplements a day. In addition, we take iron‐folic supplements, sometimes you also have to use Mebendazole and SP (sulfadoxine‐pyrimethamine for Malaria prophylaxis), you get tired, it is overwhelming’.
  IDI‐090, 34 years Primigravida
  ‘The first time I saw them (the supplements) I was shocked and I asked myself, am I going to take all these supplements? Honestly, I was scared because of the large size and quantity of supplements’.
  IDI‐110, 21 years, primigravida
  Similar remarks were also expressed by ANC health care providers that among the barriers for women to use the supplements as recommended, is the amount of the supplements, requiring them to take calcium supplements three times per day. It was explained that in addition to calcium supplements, pregnant women had several other supplements/tablets they were supposed to take:
  ‘The challenge I see these women face in taking calcium supplements every 8 hours, is because the same woman has several other tablets to take. She takes calcium …, and then she has to take iron‐folic supplements …, probably she has already swallowed SP (sulfadoxine‐pyrimethamine), and she has been given anti‐helminthic medication. She tells you it's a lot to take’.
  ANC Health care provider
  ‘Timing of swallowing calcium supplements is an issue; some women do not follow what we tell them, …. Mmh, It is a challenge for some women to take calcium supplements three times a day, every day, you know it is not a simple task especially when one is not sick’.
  ANC health care provider
iii) ***Participants’ preference for fewer calcium doses per day***

When participants were asked about their views and opinions regarding reducing the number of calcium supplements from three to one per day, they welcomed the idea very positively. They mentioned that reducing the number of calcium supplements would help women not to forget.

‘I will be easy to use because you only use it once and you are done, you cannot forget’.

IDI‐078, 30 years, multi‐gravida

Despite being in favour of reducing the number of calcium supplements per day, they voiced concerns about the health benefits if the dose is reduced. They preferred that the number of calcium supplements be reduced only if they would still get similar health benefits as with three supplements per day. Others said they would use the supplements as long as the ANC health care provider recommended. They insisted that any change to the number of supplements should be well communicated to the women with reasons for the change.

‘I think if one will still get similar benefits as we get when we use three pills, I would prefer the number of calcium supplements to be reduced’.

FGD‐06 member, 23 years, Primigravida.

‘It is not a matter of making me happy. The concern is quality and if it will help me, … So, if the nurse says that one calcium supplement per day will help me then I will take one supplement’.

IDI‐037, 24 years, Multi‐gravida

#### 3.4.3 Ethicality

Participants reported being well‐informed about the calcium supplements and their benefits during pregnancy and that their participation was a voluntary decision.

‘The nurse told me that calcium supplements do not have any problem; they are good for a pregnant woman. We were asked to use them and no one was forced; you have to use them voluntarily, that is what we were told. So, I said OK, because I want good health for my baby and myself’.

IDI‐037, 24 years, multi‐gravida

‘According to her [clinic nurse] information, I felt good because they did not just give it to me and tell me to go and swallow; they gave it to me and counselled me saying the supplements will help me and my baby’.

IDI‐090, 34 years, primigravida

#### 3.4.4 Intervention coherence

Participants expressed good knowledge of calcium supplementation following the health education from ANC health care providers. It was recognised that calcium supplements were not medicines, but rather nutritional supplements to support pregnant women with inadequate dietary calcium intake.

‘It is food, not medicines, she [the clinic nurse] said that I need to use calcium supplements to help subsidise other food that I may not get, or not afford, so those supplements contain calcium to help increase the calcium needed in the body for a pregnant woman’.

IDI‐110, 21 years, primigravida

Participants further reported being informed that the calcium supplements would help them and their babies to stay healthy. They were informed that using calcium supplements during pregnancy was beneficial in strengthening bones and preventing pre‐eclampsia and pre‐term delivery

‘They told me to pay attention to it because calcium supplements are good for the baby and the mother. They are important, so why not take them? They said calcium supplements prevent diseases or give birth to a premature baby’.

IDI‐078, 30 years, multi‐gravida

#### 3.4.5 Opportunity cost

Participants expressed that their participation in the calcium implementation study and refill of the calcium supplements were conducted during routine ANC visits. Participants reported that they were given calcium supplements free of charge and did not incur any additional costs due to participation or using calcium supplements.

‘The supplements are enough for a month. But there is one extra box in case you have an emergency and you are unable to attend the clinic on time, then you will have an extra box of supplements to continue to use’.

IDI‐092, 21 years, primigravida

#### 3.4.6 Perceived effectiveness

All participants perceived that using calcium supplements during pregnancy benefits both the mother and the baby. It was reported that calcium has helped them a lot in improving their pregnancy experiences by alleviating nausea, and vomiting and improving their appetite.

‘These calcium supplements have helped me a lot because earlier, I was not able to do anything. I was just sleeping and other people had to do everything for me … but after I came here [at the health facility] and was given these supplements, I am thankful to God, I gained energy and until now, I am doing all my activities as normal’.

FGD‐04, 23 years, multi‐gravida

‘Before I started using calcium, I was vomiting, I was not eating well, I did not like to eat or do anything, I was just lying. But when I started taking calcium supplements, my energy came back, now I eat anything I want, no more nausea. I appreciate these supplements’.

FGD‐03, 27 years, multi‐gravida

#### 3.4.7 Self‐efficacy

This construct explored the confidence of the participants to continue using calcium supplements. Despite the reported challenges in using calcium supplements, participants reported that the health education they received at the clinic regarding the importance of calcium supplements and the feeling of being responsible for the health and well‐being of the baby were the motives behind the women's determination to continue using calcium supplements.

‘I know what I am carrying, I must use calcium because I want my baby to be safe’.

FGD‐01, 41 years, multi‐gravida

‘When I look at my condition [pregnancy], I remember what I have been told at the clinic and the supplements I was given to use. So, I must use them without missing’.

FGD‐06, 24 years, multi‐gravida

## 4 DISCUSSION

This study assessed pregnant women's and antenatal health care providers' acceptability of the WHO‐recommended high‐dose calcium supplementation (three times daily with meals) for the prevention of pre‐eclampsia and its complications during pregnancy. Although participants affirmed positive attitudes towards calcium supplements, they also reported concerns about several barriers to the effective use of calcium supplements during pregnancy including large size and the burden of taking three calcium supplements per day in addition to iron ‐folic acid supplements. As a result, adherence to high‐dose calcium supplementation was found to be suboptimal.

The positive attitudes expressed by pregnant women were informed by their belief that the supplements were good for both the mother and baby's health. Similar observations of positive affirmation were also reported in Kenya, where anticipated and experienced benefits informed participants' feelings about using calcium supplements in routine ANC visits (Martin et al., 2018). However, these findings differ from those reported in Egypt, where pregnant women had negative attitudes towards calcium use (Goda et al., 2018; Shaban et al., 2020). The variations could be due to the differences in the timing of assessments. Studies in Egypt assessed acceptability prospectively (among pregnant women who had not yet used calcium), whereas in our study acceptability was assessed concurrently (among pregnant women using calcium supplements). It was noted in our study that participants reported positive attitudes about calcium supplements after they had personal experiences using them. This suggests that the acceptability of intervention is a dynamic phenomenon that is likely to change with people's experience.

Despite participants' self‐reported acceptability of the calcium supplements in this study, adherence as assessed by pill count was suboptimal with only 24.0% of the participants taking ≥90% of the calcium supplements they should have taken during pregnancy. Most participants expressed concerns about the large size of the calcium supplements and the different ways they used to overcome this challenge including crushing the supplement into two halves, chewing or dissolving it in liquids. Difficulties in taking calcium supplements due to their physical appearance have also been previously reported in Sub‐Saharan Africa (Sekhon & Van Der Straten, 2021). Therefore, demonstration of the techniques to make swallowing easier (Schiele et al., 2014) has the potential to lessen the difficulties and may improve acceptability.

The number of calcium supplements required to be taken per day was seen as a burden to pregnant women. The same pregnant women also had to take iron‐folic supplements and other interventions such as sulfadoxine‐pyrimethamine for presumptive treatment of malaria during pregnancy, mebendazole for hookworm infestation and some antiretroviral medications. Both pregnant women and antenatal health care providers stressed the challenge of adhering to a dosing schedule of taking calcium supplements three times per day throughout pregnancy. Even with good ANC attendance, adherence to nutritional supplements during pregnancy is generally a challenge (Lester et al., 2022), with a high burden of supplements being one of the factors affecting adherence and intake of oral nutritional supplements (Gomes et al., 2020).

Participants in our study expressed their preference for reducing the doses of calcium supplements to be taken per day. A recent publication of two individually randomised clinical trials by our group has demonstrated that a single low‐dose calcium supplement (500 mg/day) is as effective as a WHO‐recommended high‐dose calcium supplementation regimen (1500 mg/day) in reducing the risk of pre‐eclampsia in India and Tanzania (Dwarkanath et al., 2024). These findings address the concerns raised by some of the pregnant participants and health care providers regarding reducing the number of doses without compromising the health benefits. If adopted, a single low‐dose calcium supplementation would reduce the pill burden on pregnant women. Nonetheless, pregnant women would be advised to take at least two supplements a day—IFA or multiple micronutrient supplements (MMS) and a calcium supplement which may still pose some degree of pill burden to pregnant women. The ideal scenario would be a combined tablet to have one supplement a day or if at least that MMS and calcium could be taken at the same. Given the concerns about interaction between calcium and iron (Abioye et al., 2021), further research is needed to help understand the magnitude of the effects of interaction against the potential effects of non‐adherence.

Calcium supplements were perceived to be effective in protecting mothers from pregnancy‐related illness and their baby's health. Such perceptions were the source of motivation and commitment to continue using calcium supplements. Perceptions of benefits as a motivation for calcium supplementation use were also reported in Burkina Faso (Jones et al., 2021). Considering the importance of supplements in improving maternal health and birth outcomes (Xiang et al., 2022), building on women's perceived benefits will be critical for improving acceptability and adherence to calcium supplements.

Integrating calcium supplementation in ANC services appeared to be an acceptable approach for women since they did not have to incur extra visits or costs to access the supplements. Health services integration is acknowledged for its potential to improve effectiveness and efficiency in health care services delivery in the context of limited (Rocks et al., 2020). Integration of calcium supplementation into the current ANC programme may specifically enhance women's access to services, and continuity of care (Trankle et al., 2019). Women reported having easy access to calcium supplementation, when refills were expected during their routine scheduled ANC visits. Participants also expressed determination to continue using calcium supplements despite the efforts needed to swallow them. High self‐efficacy among participants was informed perceived benefits of using the supplements. According to Bandura (2001), the efforts people make in taking up an intervention will translate to uptake and resilience when faced with challenges. The stronger the perceived self‐efficacy, the more the effort and the higher the likelihood that a particular task would be completed.

To our knowledge, our study is the first in Tanzania to assess the acceptability of calcium supplementation during pregnancy from both women's and health care providers' perspectives. Combining quantitative and qualitative data collection methods enabled a broader and deeper understanding of women's perspectives and experiences regarding calcium supplementation during pregnancy. The theoretical framework of acceptability also helped to organise and explain the study findings andenabled the identification and description of different facets for a deeper understanding of the women's experiences and acceptability of calcium supplements. Nevertheless, we likely overestimated adherence to supplementation due to the use of pill counts and assumed that pregnant women who did not return a supplement box used all the supplements. In addition, IFA supplements were provided as standard of care by study clinics and our study emphasised assessment of calcium supplement adherence. Therefore, IFA adherence data were not available. Future studies could evaluate the relationships between calcium and IFA supplementation adherence patterns. Our findings may be generalisable only to settings of similar contexts. In addition, the study assessed acceptability concurrently. A better approach would have been to explore women's acceptability before their engagement, followed by phases of learning and assessment during and after their engagement with the supplementation for a more comprehensive understanding of how the observed acceptability may have evolved.

## 5 CONCLUSION

This mixed methods study provides useful insights into the use and acceptability of calcium supplementation from the perspectives of pregnant women and health care providers in Tanzania. Although participants had positive attitudes and expressed high self‐efficacy towards calcium supplementation, actual adherence as measured through pill count was suboptimal. Concerns about the size and number of calcium supplements were significant barriers to calcium use among pregnant women. Scaling up calcium supplementation in pregnancy should build on the existing acceptability and adherence data. Reducing the number of calcium supplement doses per day may reduce implementation costs, improve adherence and consequently lead to greater impact.

## AUTHOR CONTRIBUTIONS

Alfa Muhihi, Christopher R. Sudfeld, Mary Mwanyika‐Sando, Christopher P. Duggan, Honorati Masanja, Blair J. Wylie, Andrea B. Pembe and Wafaie Fawzi conceptualised the study. Alfa Muhihi, Emmy O. Metta and Anna Kaale participated in data collection. Emmy O. Metta and Anna Kaale conducted analysis of qualitative data. Heavenlight A. Paulo and Alfa Muhihi conducted analysis of quantitative data. Emmy O. Metta, Alfa Muhihi, Christopher R. Sudfeld and Wafaie Fawzi conducted review and interpretation of the results. Emmy O. Metta drafted and critically reviewed the manuscript. Heavenlight A. Paulo, Anna Kaale, Nandita Perumal, Mary Mwanyika‐Sando, Ndeniria O. Swai, Christopher P. Duggan, Honorati Masanja, Blair J. Wylie and Andrea B. Pembe reviewed and edited the manuscript. All authors reviewed and approved the final version of the manuscript for publication.

## CONFLICT OF INTEREST STATEMENT

The authors declare no conflict of interest.

## Data Availability

The data that support the findings of this study are available from the corresponding author upon reasonable request.
